# Supplementary material for: AlphaFold Blindness to Topological Barriers Affects Its Ability to Correctly Predict Proteins’ Topology
Source: Molecules. 2023 Nov 7;28(22):7462. doi: 10.3390/molecules28227462 (PMC10672856; doi:10.3390/molecules28227462)
Supplement: Supplementary file 1 [file molecules-28-07462-s001.zip › molecules-2557699-supplementary.pdf]

AlphaFold blindness to topological barriers affects its ability to correctly predict proteins' topology - Supplementary Material

Pawel Dabrowski-Tumanski, Andrzej Stasiak

November 3, 2023

Contents

S1 The protein models studied . . . 2
S2 The influence of the linker . . . 3
S2.1 Influence of the linker composition . . . 3
S2.2 Influence of the linker length . . . 4
S3 Alignment of sequences used in YibK loop mutants . . . 5
S4 The proximity of the modeled domains . . . 6
S5 AlphaFold structures for ATC and OTC proteins . . . 7

S1 The protein models studied

Original pdb code	Multiplicity	Linker	Topology	pLDDT	Algorithm
2efv	5x	GGGGGGGGG	5x 3.1	78.71649006669325	monomer
2efv	10x	GGGGGGGGG	10x 3.1	77.70888245659702	monomer
2efv	1x	-	3.1	89.43370110103803	monomer
2efv	2x	G	2x 3.1	86.99612401234245	monomer
2efv	3x	GGGGGGGGG	3x 3.1	82.8152655055198	monomer
2efv	3x	P	3x 3.1	85.81011294788604	monomer
2efv	3x	PPPPPPPPP	3x 3.1	81.23540016817718	monomer
1j85	3x	G	3x 3.1	85.20678834512394	monomer
1j85	3x	GGGGGGGGG	3x 3.1	76.88002276812513	monomer
1j85	3x	SGSGSGSGS	3x 3.1	80.2483009896914	monomer
1j85	2x	G	2x 3.1	87.73989010514727	monomer
1j85	2x	GGGGGGGGG	2x 3.1	87.49120111818907	monomer
1j85	2x	SGSGSGSGS	2x 3.1	87.81138046229104	monomer
1j85	5x	GGGGGGGGG	5x 3.1	78.39719131243979	monomer
1j85	1x	-	3.1	89.13466474291562	monomer
1j85	1x	-	3.1	89.21932363785083	monomer
3kzn	1x	-	3.1	93.8598730676699	monomer
4jqo	1x	-	0.1	92.59100835981404	monomer

Table S1: The list of protein models studied along with their linkers, topology, algorithm, and pLDDT of the best model. For the model structures and sequences please see `https://gitlab.com/pdabrowskitumanski/alphafoldwrapper`

S2 The influence of the linker

Usually, the domains were connected by a flexible linker composed of 9 glycine residues. However, we checked the influence of linker composition and linker length on the modeled structures.

S2.1 Influence of the linker composition

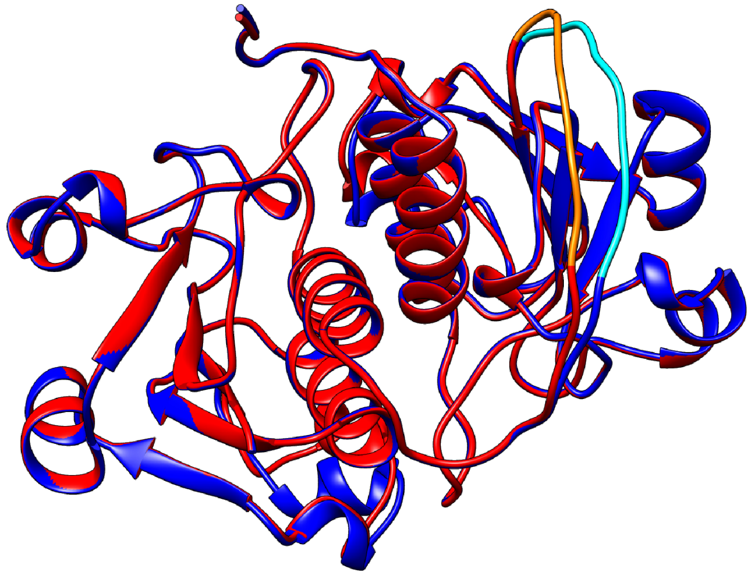

Figure S1: Comparison of tandem repeats of two copies of YibK domains (PDB code 1j85). Red structure with orange linker 2x1j85 with GSGSGSGSG linker (9 residues), blue structure with cyan linker 2x1j85 with GGGGGGGGG linker (9 residues). As can be seen, the difference may be found only in the linker and long, unstructured tails.

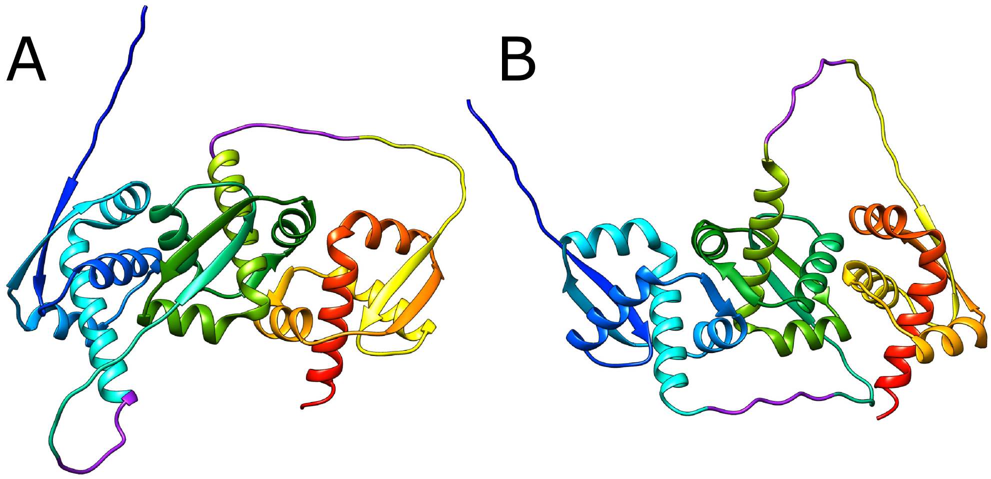

Figure S2: Comparison of tandem repeats composed of three copies of MJ0366 domains (PDB code 2efv) and having different linker length. A) 3x2efv with GGGGGGGGG linker (9 residues), B) 3x2efv with PPPPPPPPP linker (9 residues). The linkers are marked with a violet color. The structures are not superimposable, as the relative orientation of domains differs. However, the structure and topology of each domain remain unchanged. The difference can be seen in the linkers, where the stiffer proline-rich piece of the chain forces different linker arrangements.

S2.2 Influence of the linker length

Both starting structures (PDB codes 2efv and 1j85) feature long, unstructured tails that add up to the linker. We checked, therefore, if the linker length influences the structure and topology of tandem repeats.

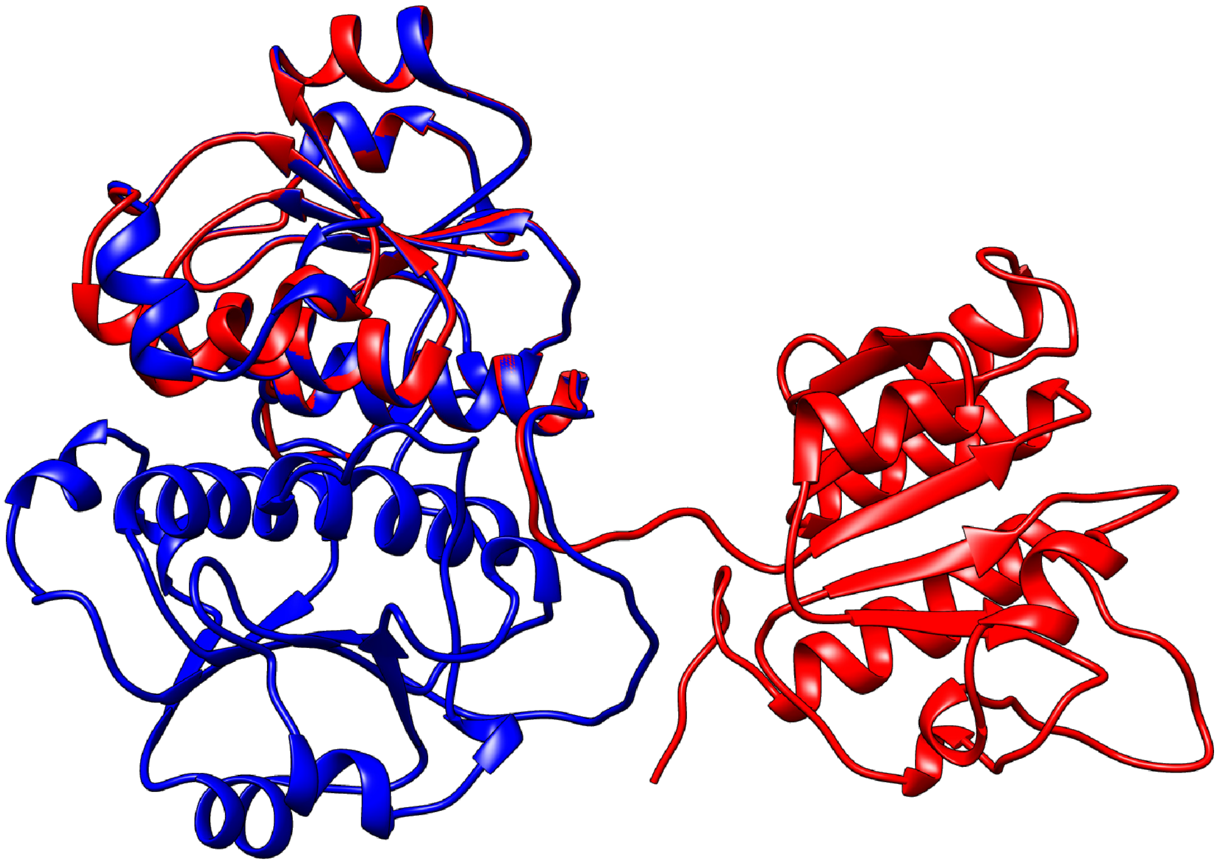

Figure S3: Comparison of tandem repeats composed of two copies of YibK domains (PDB code 1j85) differing in linker length. Red structure - linker composed of a single Glycine residue. Blue structure, linker composed of 9 Glycine residues (GGGGGGGGG). In both structures each domain is superimposable, also the topology does not change. However, the mutual location of the domain differs. In the structure with the longer linker (blue), the domains remain spatially close.

S3 Alignment of sequences used in YibK loop mutants

WT (1-50)	MLDIVLYEPEIPQNTGNIIRLCANTGFRLHLIEPLGFTWDDKRLRRSGLD
Mutant 1 (1-50)	MLDIVLYEPEIPQNTGNIIRLCANTGFRLHLIEPLGFTWDDKRLRRSGLD
Mutant 2 (1-50)	MLDIVLYEPEIPQNTGNIIRLCANTGFRLHLIEPLGFTWDDKRLRRSGLD
WT (51-100)	YHEFAEIKRHKTFEAFLESEKPKRLFALTTKGCPAHSQVKFKLGDYLMFG
Mutant 1 (51-100)	YHEFAEIKRHKTFEAFLESEKPKRLFALTTKGCP-----KFKLGDYLMFG
Mutant 2 (51-100)	YHEFAEIKRHKTFEAFLESEKPKRLFALTTK--P-----KFKLGDYLMFG
WT (101-150)	PETRGIPMSILNEMPMEQKIRIPMTANSRSMNLSNSVAVTVYEAWRQLGY
Mutant 1 (101-150)	PETRGIPMSILNEMPMEQKIRIPMTANSRSMNLSNSVAVTVYEAWRQLGY
Mutant 2 (101-150)	PETRGIPMSILNEMPMEQKIRIPMTANSRSMNLSNSVAVTVYEAWRQLGY
WT (151-160)	KGAVNLPEVK
Mutant 1 (151-100)	KGAVNLPEVK
Mutant 2 (151-100)	KGAVNLPEVK

Table S2: The alignment of mutants of YibK protein (PDB structure 1j85) differing in the loop size and composition. Each of the mutants is modeled by AlphaFold as a 3_1-knotted protein. In the main text, Mutant 2 is presented.

S4 The proximity of the modeled domains

Some protein structures present in the RCSB database exist as biological assemblies, where more than one domain is present. In particular, both structures (with PDB codes 2efv and 1j85) exist as homodimers in their assemblies. The information, about the spatial proximity of the domains in the original structure is passed through the AlphaFold algorithm placing the domains in the tandem repeats spatially close if possible.

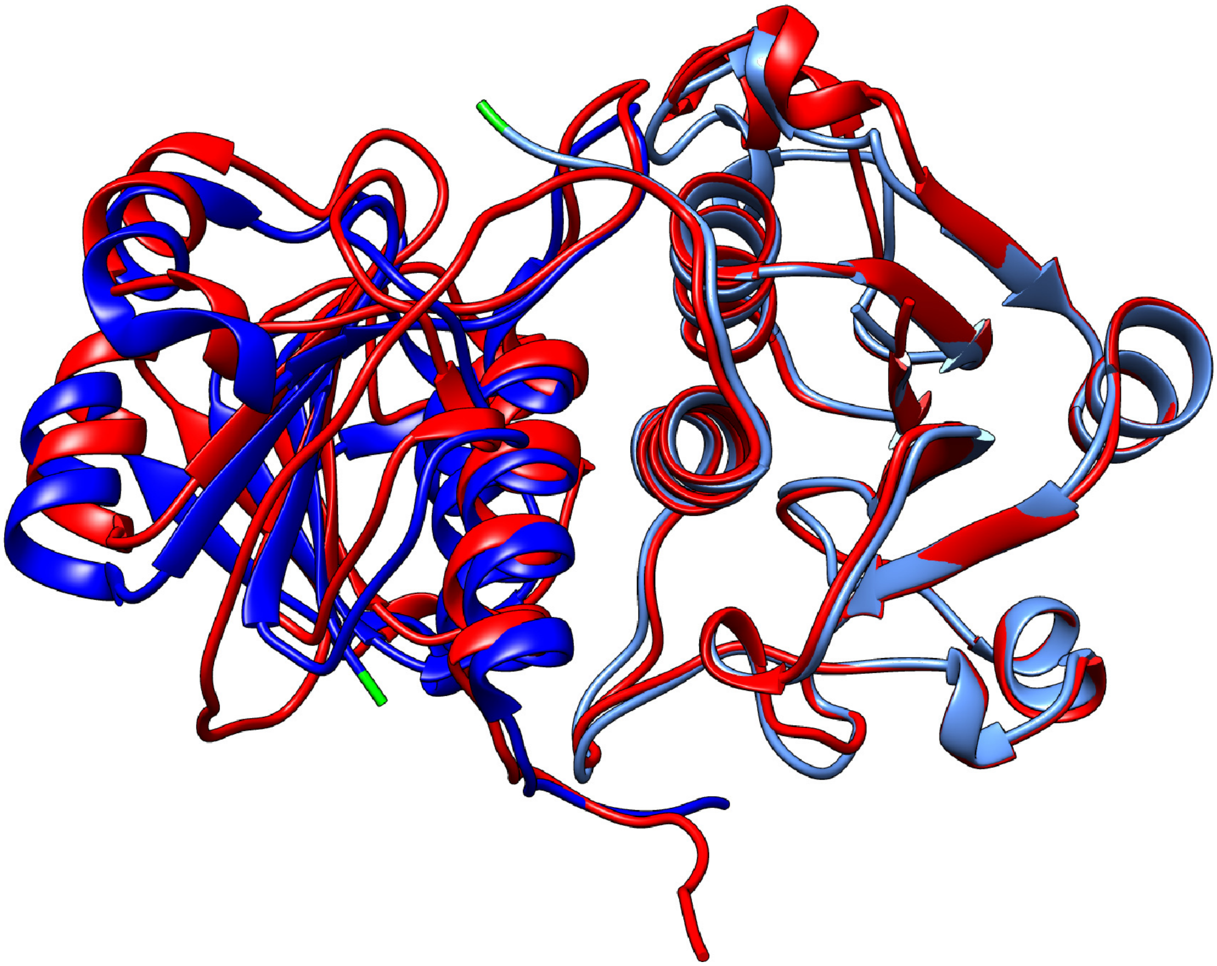

Figure S4: Comparison of the biological assembly of YibK (PDB code 1j85) with AlphaFold model of 2x1j85 with linker GGGGGGGGG (9 residues). The AlphaFold model is marked with red, the original assembly in two shades of blue, each individual chain in a different shade. The blue domains are not covalently connected. The end of the first chain and the start of the second chain in the assembly are marked with green. These residues are connected in the model by the linker. The spatial proximity of the domains does not influence the topology, which is 3_1 for each domain.

S5 AlphaFold structures for ATC and OTC proteins

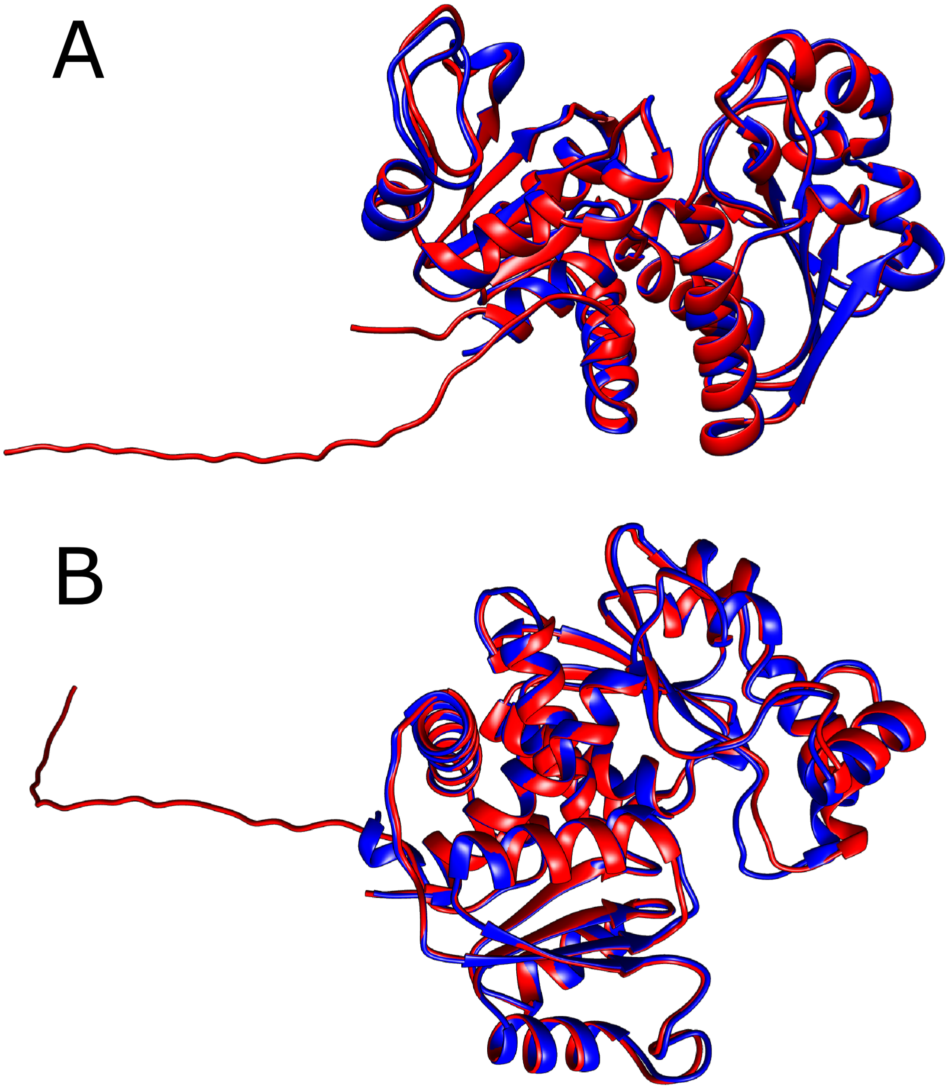

Figure S5: Comparison of crystal structures of N-acetyl-L-ornithine transcarbamoylase (ATC - 3_1-knotted, panel A) and ornithine transcarbamoylase (OTC - unknotted, panel B) with their models obtained by AlphaFold. The original structures are depicted in blue. The models are depicted in red. In both models, the long unstructured tail missing in the original crystal structure was modeled. The structures are superimposable in both cases with only minor differences. In particular, AlphaFold finds the correct topology in both cases.
